# Improving Teletherapy Enrollment Using Quality Improvement Methodology for Psychiatry Patients Boarding on Acute Care Services

**DOI:** 10.1097/pq9.0000000000000872

**Published:** 2026-02-20

**Authors:** Katherine A. Ritter, Mahdieh Bodaghi, Padmaja Pavuluri, Stacey Stokes

**Affiliations:** 1 From the Department of Hospital Medicine, Children’s National Medical Center, Washington, DC.

## Abstract

**Introduction:**

In 2021, the American Academy of Pediatrics, the American Academy of Child and Adolescent Psychiatry, and the Children’s Hospital Association declared a state of emergency in children’s mental health. Responding to this emergency, our freestanding children’s hospital began a novel teletherapy program to provide teletherapy services to patients admitted to acute care units while awaiting inpatient psychiatric care. The global aim of this quality improvement project was to improve access to inpatient teletherapy. The specific aim was to increase the number of patients who completed at least 1 teletherapy session from a median of 2–4 per month and sustain this increase for 3 months.

**Methods:**

Using the Model for Improvement framework, multidisciplinary stakeholders created process maps and Ishikawa diagrams, followed by key driver diagrams, to outline areas for improvement in the teletherapy enrollment process. Interventions included staff education, improved communication techniques, and enhancements to the electronic health record (EHR). We implemented interventions as Plan-Do-Study-Act cycles, collected data via a custom EHR report, and analyzed data using a run chart.

**Results:**

After completing 3 Plan-Do-Study-Act cycles, the median number of patients with teletherapy orders increased from 2 to 4 per month, a level we sustained for 5 months.

**Conclusions:**

Implementing and sustaining a novel group teletherapy workflow on acute care units is a multidisciplinary and complex process that requires multistakeholder coordination. We achieved a sustained increase in teletherapy enrollment through staff engagement, team member education, and EHR clinical decision support.

## INTRODUCTION

In 2021, the American Academy of Pediatrics, the American Academy of Child and Adolescent Psychiatry, and the Children’s Hospital Association declared a state of emergency in children’s mental health.^1^ With pediatric depression and anxiety diagnoses increasing steadily over the last several decades, the COVID-19 pandemic accelerated the crisis due to a confluence of social isolation and a lack of accessible services.^2^ Before the pandemic, 13% of adolescents received some form of school-based mental health services, and 35% of adolescents relied exclusively on these services.^3^ During the pandemic, school shutdowns and medical home closures limited access to critical mental health resources. Meanwhile, loneliness is known to correlate with depressive symptoms, and social isolation is considered a risk factor for loneliness.^4^

During the pandemic, the proportion of mental health–related emergency department (ED) visits increased by 31% among adolescents aged 12–17 years from 2019 to 2020.^5^ Additionally, the prerequisite for a negative COVID-19 test for inpatient psychiatric unit admission prolonged ED lengths of stay for patients awaiting admission.^6^ Patients boarding in the ED, defined as “the practice of holding patients in the emergency department or in another temporary location after the decision to admit or transfer has been made,”^7^ increased by 76% compared with prepandemic levels.^8^

Inpatient boarding for patients with primary mental health diagnoses increased as well. A large retrospective study among tertiary children’s hospitals found that from 2017 to 2023, the median boarding length of stay in the ED and/or inpatient units increased from 3 to 4 days, with the number of boarding encounters peaking in 2021.^9^ There is a paucity of literature addressing ways to improve the experience of children and adolescents boarding on inpatient acute care units (ACUs) while awaiting placement in an inpatient psychiatric unit.^10^ Due to mental health safety concerns, these patients are often isolated and confined to their rooms and receive minimal mental health services while boarding. Meanwhile, the financial, emotional, and cognitive burdens placed on patients and medical teams have been well documented.^11^

Early in the COVID-19 pandemic, all patients in our hospital requiring inpatient psychiatric admission who incidentally tested positive for COVID-19 were required to board on ACUs until the completion of their isolation period. Additionally, during the pandemic, the inpatient psychiatry unit’s capacity decreased by half as double rooms were converted to single rooms for infection control. In this context, our hospital began a novel ACU teletherapy program, designed to leverage the hospital’s existing resources to expand mental health treatment for boarding patients without placing additional strain on an already-overextended system.

We applied the Institute for Healthcare Improvement’s Model for Improvement to enhance the implementation of this program. The program uses a secure meeting platform (Zoom Video Communications, San Jose, CA) on a wireless tablet to connect ACU patients to existing in-person group therapy sessions on the hospital’s adolescent psychiatric inpatient unit (APU) and child psychiatric inpatient unit. These units are part of a separately licensed inpatient psychiatric facility located within the same building. Enrollment criteria included behavioral health patients requiring inpatient psychiatric care, medical clearance, the presence of a one-on-one safety observer in the room, admission to 1 of 2 designated ACUs, and a recommendation by the psychiatry consult team for participation in teletherapy. Enrollment relied on complex, multidisciplinary coordination and communication between multiple stakeholders and units, averaging 2 patients per month. This project’s global aim was to improve access to mental health care for patients boarding on ACUs awaiting psychiatric placement, increasing the value of their hospitalization. Our specific aim was to increase the median number of qualifying patients completing at least 1 teletherapy session from 2 to 4 per month and sustain this increase for 3 months.

## METHODS

### Context

We conducted this project at an urban, freestanding, academic children’s hospital with 323 inpatient pediatric beds and approximately 125,000 ED patient visits per year. ACU services (hospital medicine and adolescent medicine) admit patients who require medical stabilization related to a psychiatric diagnosis. These services have a combined average daily census of approximately 55 patients. Once medically cleared, the psychiatry consult team evaluates the patient to determine the appropriate disposition. If a patient is deemed appropriate for inpatient psychiatric admission but no inpatient psychiatric beds are available, the patient remains on a medical service until a bed becomes available.

From March 2020 through February 2021, the average length of stay at the hospital for a patient boarding on an ACU due to a psychiatric diagnosis was 11 days. In that time, 2 patients required eventual transfer to inpatient psychiatry once a bed was available. Two patients required medical and physical restraints at some point during their ACU admission. From January 2022 to December 2023, there were 241 patient days, averaging 10 patient days per month, during which patients with primary psychiatric diagnoses were boarding on ACUs.

### Design and Interventions

The primary investigator received project funding in the form of protected time for 18 months during residency training. We defined teletherapy enrollment as the placement of a teletherapy order in our electronic health record (EHR) (Cerner PowerChart, Oracle Health, Kansas City, MO), which was necessary for nursing staff to implement the teletherapy protocol. The institutional review board determined this quality improvement (QI) project to be non–human subjects research and therefore exempt from review due to the de-identified nature of the data.

**Fig. 1.**
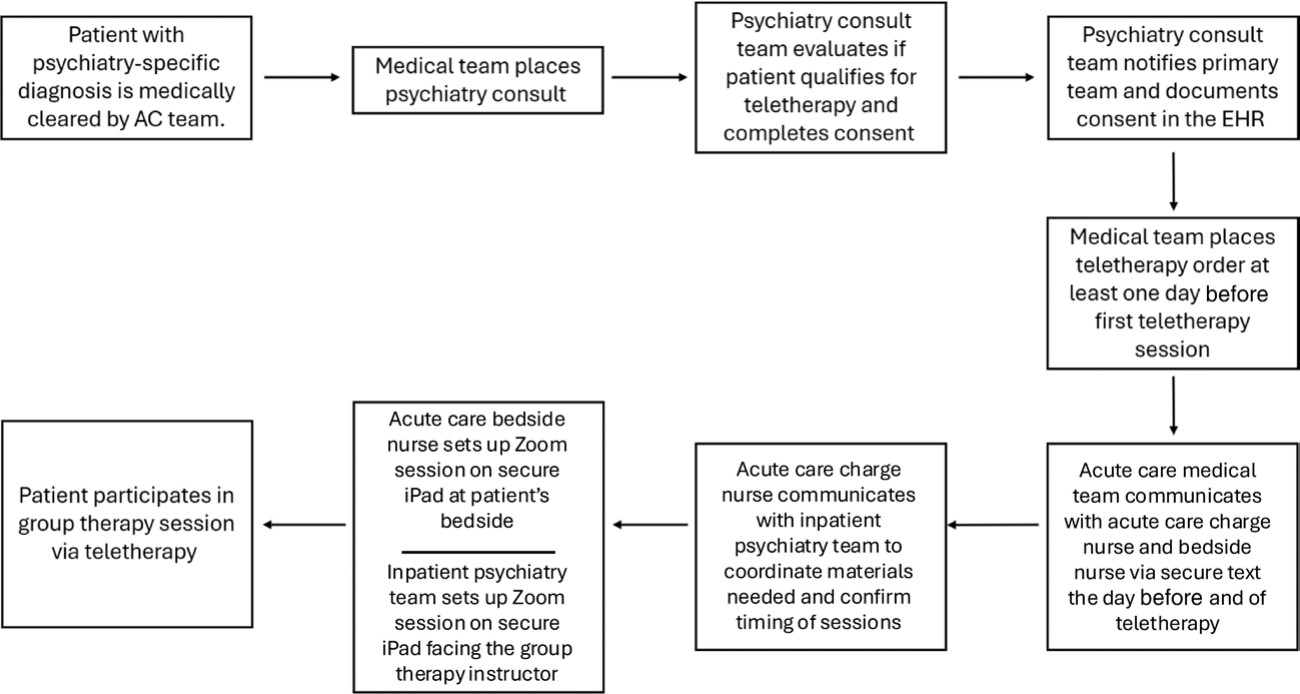
Process map.

**Fig. 2.**
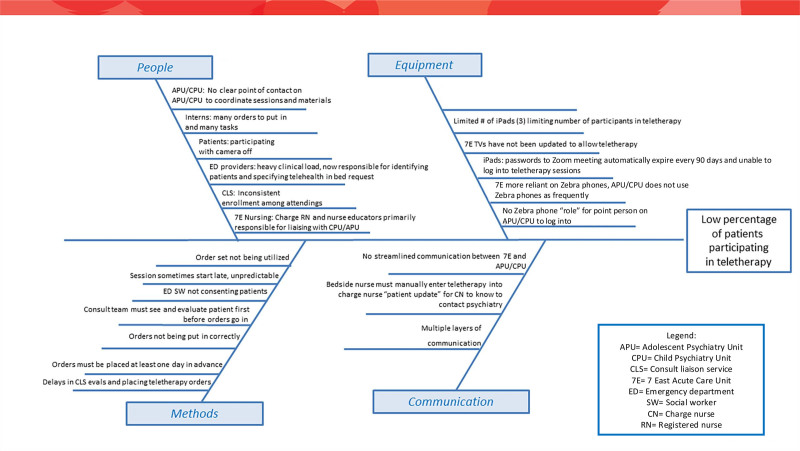
Ishikawa diagram.

**Fig. 3.**
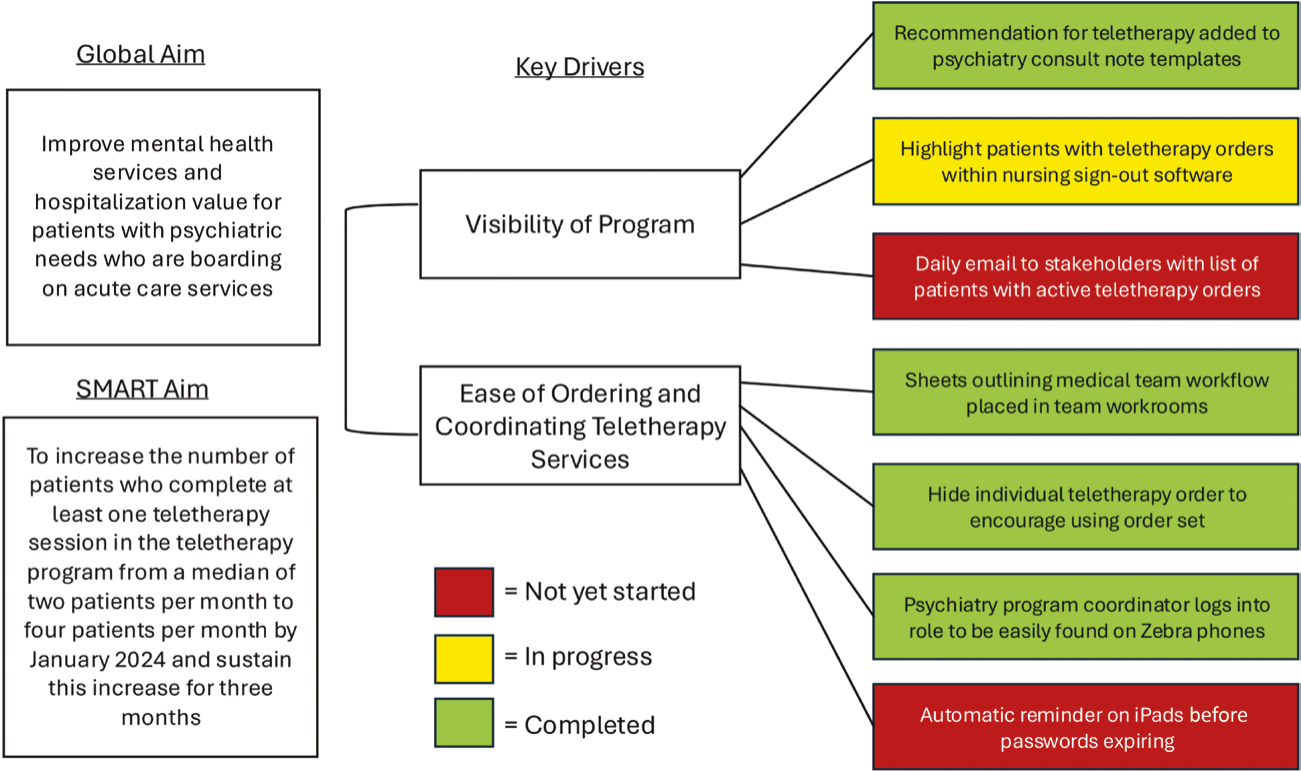
Key driver diagram.

We conducted a problem analysis with multidisciplinary stakeholders, including ACU nursing leadership, psychiatry nursing leadership, and inpatient and consulting psychiatry and hospital medicine attending physicians. We held 3 large-group, open forum–style meetings during a 3-month period. Stakeholders outlined the teletherapy enrollment workflow on a process map (Fig. 1), diagrammed the problem analysis on an Ishikawa diagram (Fig. 2), and created a key driver diagram (Fig. 3) targeting program visibility and streamlined ordering and coordination as primary areas for improvement.

### Data Monitoring

As a proxy for the number of participating patients, the outcome measure was the number of patients per month with a teletherapy order, captured by a custom EHR report. This approach replaced a prior labor-intensive collection process that resulted in missing patients if they were not manually added to the patient tracker.

**Fig. 4.**
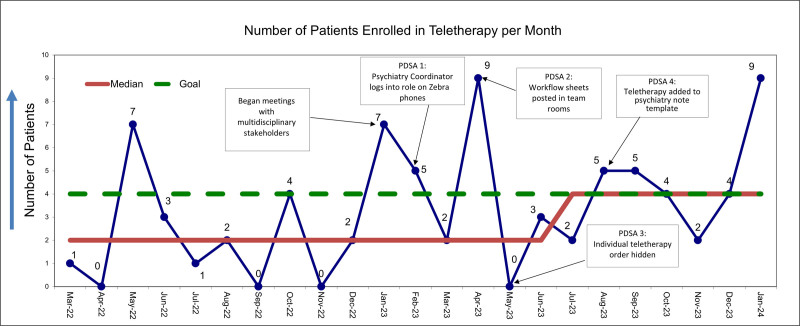
Run chart. CPU, child psychiatric inpatient unit.

The study team plotted the number of patients with a teletherapy order per month on a run chart (Fig. 4). We elicited feedback after Plan-Do-Study-Act (PDSA) cycles in large-group meetings with multidisciplinary stakeholders. Additionally, the project’s consultant psychiatrist provided real-time feedback on the teletherapy implementation process at project meetings, which we held approximately every 3 months. The project’s primary investigators held project meetings every other month to review data and discuss progress.

As there is no requirement in the EHR to automatically document when a patient completes a teletherapy session, we used chart review as a proxy validation strategy. One study team member (K.A.R.) manually reviewed the charts of all patients plotted in the run chart to validate whether a teletherapy session had occurred. We considered teletherapy completion to be validated if documentation in a progress note, consult note, discharge summary, or nursing note indicated that a patient consented to, participated in, or was planning to participate in teletherapy. This confirmatory documentation validates that, for the majority of patients, an order placed is an appropriate representation of the teletherapy received. Absence of such documentation does not necessarily indicate that a patient did not complete teletherapy.

### PDSA Cycles

We completed 4 PDSA cycles (see Table 1) during a 6-month period. The first PDSA cycle targeted the interunit communication between ACU nursing and inpatient psychiatry unit coordinators (PUCs) by providing PUCs access to the same mobile phones (Zebra TC5 phones, Zebra Technologies Corporation, Lincolnshire, IL) with secure text functionality (CareAware Connect Messenger, Cerner Corporation, Kansas City, MO) and role-based assignments relied on by ACU nursing staff. This approach streamlined the coordination and timing of teletherapy sessions via messaging.

**Table 1.** PDSA Cycles

PDSA Cycle	Plan	Do	Study	Act
PDSA 1	1. Determined which roles within the secure message platform (CareAware Connect Messenger) were appropriate for standardized communication 2. Identified all appropriate stakeholders in inpatient psychiatry 3. Made sure phones were available on the inpatient psychiatry unit	1. Psychiatry unit coordinators were instructed on how to log into a role-based contact number in Zebra phones 2. Policy updated to reflect this workflow change, and nurse educators on AC informed charge nurses of the change 3. Charge nurses began texting psychiatry unit coordinators through CareAware Connect Messenger when a patient was enrolled in teletherapy	Initial feedback was that psychiatry unit coordinator role assignment rates were low. The team met with coordinators to troubleshoot several barriers to use, and monitoring continued	Psychiatry unit coordinator self-assignment improved during 3–4 mo, and nursing staff on AC communicate through CareAware Connect Messenger to inpatient psychiatry when a patient is enrolled
PDSA 2	Resident physicians were queried on their knowledge of the teletherapy program and expressed confusion on how to order teletherapy, how to determine which patients should receive therapy, and how to assist nursing staff in facilitating teletherapy sessions	Easy-to-read flow sheets outlining expectations and responsibilities of resident physicians were posted in all inpatient team rooms	After 2 mo, the median shifted from 2 to 4 patients per month Highest enrollment was achieved (9) in the first month of this intensive education	Residents were regularly reminded that these sheets are posted in team rooms and contain many answers to residents’ frequently asked questions
PDSA 3	The individual teletherapy order was still visible in the EHR outside of an order set, causing confusion on which orders were needed	The individual teletherapy order was hidden from view so that teletherapy could only be ordered using the teletherapy order set	After 1 mo, the median shifted from 2 to 4 patients per month	The individual teletherapy order remains hidden, ensuring that all necessary orders regarding timing and materials are ordered each time a patient is ordered for teletherapy
PDSA 4	Improve visibility of the teletherapy program to the consult psychiatry team through clinical decision support	The phrase “please order patient for group teletherapy” was added to psychiatry consult template notes	The median shift from 2 to 4 patients per month was sustained more than 3 mo	This phrase has been added to many psychiatry attendings’ consult notes, framing teletherapy as a standard inpatient treatment for all eligible patients

The second PDSA cycle targeted pediatric resident physicians, the frontline providers responsible for placing teletherapy orders, and aimed to increase their understanding of teletherapy workflow. The study team posted information sheets outlining the role of resident physicians in patient enrollment in all resident physicians’ workrooms.

Feedback, however, cited ongoing confusion about the teletherapy ordering process. The next intervention leveraged EHR solutions to streamline the ordering process. During the initial pilot, the study team worked with hospital information technologists to create a new teletherapy order set. This order set automated all required orders, including the teletherapy order itself, with auto-populated dates and times, as well as the necessary nursing communication orders. However, physicians could still order teletherapy by using a discrete order outside of the order set. Without the associated decision support, ordering using the individual order led to missed nursing communication and frequently incorrect teletherapy session dates and times. Thus, the third PDSA cycle hid the discrete teletherapy order, forcing users to order teletherapy sessions using the complete order set.

The fourth PDSA cycle aimed to increase visibility of the teletherapy program among the psychiatry consult team members themselves. The project team’s consult psychiatrist was also the Medical Director for the Psychiatry Consult Liaison Service, whose consult note template was widely used by other psychiatry attendings and fellows. This consult psychiatrist updated the template to include teletherapy as a recommended treatment option, elevating its use as the standard inpatient treatment for eligible patients.

## RESULTS

Baseline data from March 2022 through January 2023 showed a median monthly enrollment of 2 patients. After the third PDSA cycle and 5 months into the project, a median shift to 4 patients per month occurred. We sustained this shift 5 months after the final PDSA cycle. The most notable improvement in patient enrollment occurred after the first PDSA cycle, when the PUC began logging in to CareAware Connect Messenger. We observed sustained enrollment improvement after the fourth PDSA cycle, when the recommendation for teletherapy was added to the psychiatry note template.

We defined enrollment as the presence of a teletherapy order and then plotted the number of patients with a teletherapy order each month on a run chart (Fig. 4). Seventy-seven patients received teletherapy orders during 23 months. Per chart review, 58 (75%) patients had confirmatory EHR documentation (eg, progress notes, discharge summaries, nursing notes, or psychiatry consult notes) indicating that teletherapy sessions occurred.

Twenty-seven (35%) patients were ultimately admitted to the hospital’s APU or child psychiatric inpatient unit, with a median inpatient psychiatric length of stay of 8 days (range of 4 –56 d) and an average length of stay of 12.2 days. Of the 50 remaining patients, 29 (58%) were discharged home, and 21 (42%) were discharged to an outside mental health facility. Four (5%) of the 77 patients were readmitted to an ACU for a psychiatric chief complaint requiring medical stabilization (eg, suicide attempt or acute food refusal) within 90 days of discharge (range 8–85 d, median 34 d, and average 40.3 d): 1 was initially admitted to the hospital’s APU, 2 were transferred to an outside mental health facility, and 1 was discharged home. Although feedback from patients and families was not specifically investigated, no adverse events were reported during teletherapy by patients, family members, or clinicians.

## DISCUSSION

Implementing and sustaining a novel group teletherapy workflow on ACUs requires complex coordination across units and multiple stakeholders. By applying the QI methodology and implementing interventions targeting stakeholder engagement, staff education, and just-in-time clinical decision support (ie, an order set), we met and sustained our specific aim of increasing the median number of qualifying patients completing at least 1 teletherapy session from 2 to 4 patients per month.

Telemedicine can bridge gaps in access to care.^12^ Even before the pandemic, teletherapy was known to be a cost-effective strategy to deliver care in an already-overstretched system.^13^ Additionally, studies have shown comparable satisfaction rates between teletherapy and in-person psychotherapy in children and adolescents,^14^ similar reductions in depressive symptoms,^15^ and equivalent treatment outcomes and attrition rates.^16^ During the pandemic, parents of children and adolescents and their therapists reported high satisfaction rates with teletherapy.^17^

Wood et al^18^ found that teletherapy implementation is amenable to PDSA cycles and a QI framework. After recognizing a need for virtual solutions during the pandemic, they used QI methodology to pilot an ambulatory group teletherapy program for patients with psychosis. They reported high satisfaction rates and adherence to teletherapy, highlighting that group teletherapy can be an effective and accessible treatment modality.^18^ Another research project in Veterans Affairs clinics used PDSA cycles to expand teletherapy access in rural clinics, finding that individual therapists’ confidence in teletherapy, in part, determined its success as a treatment modality.^19^

QI methodology has also proved effective in implementing telemedicine in pediatric ambulatory settings. Vallabhan et al^20^ used the Model for Improvement framework to implement telemedicine in an ambulatory pediatric weight management clinic at the beginning of the pandemic. Like our project, this project strove to improve care delivery to an already-vulnerable population (in this case, pediatric patients with obesity) at risk of poor health outcomes in the context of the pandemic. Using PDSA cycles, they achieved a sustained increase in patient visits per week through telemedicine and improved patient satisfaction scores compared with the pretelemedicine period.^20^ Similarly, Maitre et al^21^ used QI methodology to improve care delivery to another vulnerable population: infants at risk for developmental delays who were unable to receive in-person neurobehavioral assessments and surveillance during the pandemic. Like our project, these researchers relied on stakeholder engagement to achieve their aim. They trained multidisciplinary providers to adapt their assessments to a virtual platform, resulting in high parent and provider satisfaction with telehealth and no significant difference in diagnostic accuracy between telehealth visits and a matched in-person cohort.^21^

Based on the success of these projects, we believe that, with multidisciplinary buy-in, inpatient group teletherapy could be an effective and accessible treatment modality for our ACU patient population and is amenable to process improvement using QI methodology. Given the nationwide increase in both pediatric mental health conditions and boarding times, we hope that our project’s success, achieved through stakeholder engagement, QI methodology, and limited resources, can inspire similar initiatives at other institutions.

### Limitations

There were several limitations to this project. First, the number of ACU patients eligible for teletherapy decreased as the prevalence of COVID-19 waned and COVID-19-related guidelines for psychiatric unit admissions liberalized, limiting our study size. A second limitation was the inability to capture a true denominator, that is, the total number of patients eligible for teletherapy. Thus, it is unclear whether the increase in enrollment was secondary to a larger number of eligible participants or to process improvements. Future work should focus on reliably identifying this denominator, enabling more accurate monitoring of progress.

A third limitation was the inability to auto-document teletherapy session completion in the EHR. We used manual chart review to validate teletherapy completion, relying on discretionary ad hoc documentation in psychiatry consult notes, progress notes, and discharge summaries. This process is labor-intensive and less accurate than an automated EHR notation, as there is no requirement to document the completion of a teletherapy session in the EHR. Further work includes establishing a standardized, designated method for documenting teletherapy session completion in the EHR.

### Strengths

Our study had several strengths. First, we validated actual teletherapy completion using a manual chart review. Second, our PDSA cycles were multimodal (targeting workflow, education, and EHR solutions), increasing the likelihood for sustainability. Third, the interventions involved multiple stakeholder groups (ie, psychiatry consult team, inpatient psychiatry and ACU nursing teams, and pediatric resident physicians). A large, engaged, multidisciplinary team made success possible. Stakeholders across the workflow spectrum were interactive and open to change, providing feedback throughout the process to improve performance. Finally, by using dynamic problem-solving, the team was able to quantify teletherapy involvement despite the absence of an automated patient tracker in the EHR.

## CONCLUDING SUMMARY

Early and ongoing stakeholder engagement, along with multiple successful PDSA cycles, resulted in a sustained increase in teletherapy program enrollment among qualifying patients. Multimodal PDSA cycles included technology-based solutions related to communication, clinical documentation, EHR clinical decision support, and team member involvement through targeted education strategies. This project achieved its global aim of expanding access to mental health care, improving the value of hospitalization for boarding patients awaiting definitive psychiatric care. Future work should focus on streamlining data collection and accuracy, as well as on capturing the true baseline of patients eligible for teletherapy. Future work should also include efforts to measure the clinical impact of teletherapy, including patient, family, and provider feedback on its processes and content.
